# Evaluation of pressure-controlled mammography compression paddles with respect to force-controlled compression paddles in clinical practice

**DOI:** 10.1007/s00330-018-5953-6

**Published:** 2019-01-07

**Authors:** C. R. L. P. N. Jeukens, T. van Dijk, C. Berben, J. E. Wildberger, M. B. I. Lobbes

**Affiliations:** 1 Department of Radiology and Nuclear Medicine, Maastricht University Medical Center, P. Debyelaan 25, PO Box 5800, 6202AZ Maastricht, The Netherlands; 2 Department of Medical Physics, Máxima Medical Center, Veldhoven, The Netherlands; 3 GROW School for Oncology and Developmental Biology, Maastricht, The Netherlands

**Keywords:** Mammography, Radiation dosage, Pain

## Abstract

**Objectives:**

To reduce pain and discomfort associated with breast compression in mammography, a pressure-controlled compression paddle was recently introduced.

The objective was to evaluate the pressure-controlled paddle by comparing it to the standard force-controlled paddle.

**Methods:**

Differences of compressed breast thickness (CBT), compression force, compression pressure, and average glandular dose (AGD) between annual follow-up full-field digital mammography exams of 3188 patients were retrospectively examined. Two groups were compared: (1) *force-force group* (FF-group), both examinations were performed with the force-controlled paddle, and (2) *force-pressure group* (FP-group), only the follow-up examination was performed with the pressure-controlled paddle.

In an additional group of patients, pain scores on a scale of 0 (no pain at all) to 10 (worst pain imaginable) were evaluated prospectively (*n* = 343) who were randomly assigned to either paddle.

**Results:**

Median differences between follow-up exams in CBT, compression force, compression pressure, and AGD were for the FF- and FP-group respectively − 1.0 vs 0.0 mm (*p* < 0.001); 0.0 vs − 1.0 daN (*p* = 0.002); − 1.0 vs − 0.5 kPa (*p* = 0.005); and 0.05 vs − 0.02 mGy (*p* < 0.001). These differences were, although statistically significant, clinically non-relevant (defined as ΔCBT > ± 2 mm; Δforce > ± 2 daN; Δpressure > ± 1 kPa and ΔAGD > ± 0.1 mGy). The subanalysis dividing CBT into five categories revealed similar results. The median [interquartile range] pain scores were 6 [3, 7] and 5 [3, 7] for the force-controlled and pressure-controlled paddle, respectively, which was not significantly different (*p* = 0.113).

**Conclusions:**

We observed no clinically relevant differences in CBT, compression force, compression pressure, AGD, or pain score between the force- and pressure-controlled paddle. As such, we found no basis for preferring one paddle over the other.

**Key Points:**

• *The pressure-controlled paddle did not show any clinically relevant changes in breast compression parameters compared to the force-controlled paddle*.

• *The pressure-controlled paddle did not lead to significant reduction in pain scores indicated by the patients compared to the force-controlled paddle*.

• *A large variation in compression force and compression pressure was observed in mammography exams for the both the force- and pressure-controlled compression paddle*.

**Electronic supplementary material:**

The online version of this article (10.1007/s00330-018-5953-6) contains supplementary material, which is available to authorized users.

## Introduction

Mammography is currently the worldwide routine examination for breast cancer detection. During a mammographic exam, the breast is compressed to improve image quality and to decrease radiation dose [1]. However, breast compression comes at the price of discomfort and pain. Hence, efforts have been undertaken to reduce this drawback, such as pain medication prior to the exam, providing patients with information, breast cushions, and curved paddles [2, 3].

Recently, a new method was developed using a pressure-controlled compression paddle [4, 5]. In standard practice, a force-controlled paddle compresses the breast until a target force is reached, irrespective of the breast size. The pressure-controlled paddle compresses the breast until a target pressure is obtained. The pressure is determined as the force exerted divided by the breast-paddle contact area. Thus, compressing until a target pressure leads to a lower compression force for smaller breasts having a smaller contact area than for larger breasts. This would reduce discomfort and pain for the smaller breasts, as it has been shown that the tolerance of compression force decreases with decreasing contact area [5, 6].

Previous studied showed [4, 7] that the pressure-controlled paddle resulted in a more standardized compression pressure, a slightly increased compressed breast thickness (CBT), and lower pain experience without affecting image quality or increasing the average glandular dose (AGD). These studies were performed in an experimental setting using a well-defined compression protocol in which the technician was instructed to compress until a customized display indicated “100% compression,” being either the target force or pressure, for which the technician was blinded.

However, it is known that in everyday clinical practice, being unblinded for the equipment used and less strictly protocolized, compression force varies considerably [8, 9]. The aim of this study was to evaluate the pressure-controlled paddle in the everyday clinical practice of our institution. In case the pressure-controlled paddle would lead to pain reduction, this would be reflected in changes of objective parameters such as CBT, compression force and pressure, and AGD. Therefore, primary study outcomes were differences in these parameters of subsequent follow-up exams using either both force-controlled paddles or first time force-controlled, second time pressure-controlled paddle. Furthermore, a primary study outcome was the subjective pain score of an examination using either the pressure- or force-controlled paddle.

## Materials and methods

### Study population

FFDM exams performed at our institution for annual follow-up (indications, e.g., family predisposition, BRCA1/2 gene mutation carriers, breast cancer history) were retrospectively retrieved from the Picture Archiving and Communication System (PACS) (period 01/2014–12/2016). All examinations were randomly assigned to either of the two identical mammography units (Senographe Essential, GE Healthcare) undergoing equal quality control testing twice a year. Consecutive follow-up examinations form a so-called follow-up examination pair. CC-views of both breasts, or a single CC-view in case only one breast was examined, were included. Inclusion criteria were time between follow up ≤ 24 months; BI-RADS score was 1 (negative) or 2 (definitely benign), and the same for both follow-up exams (i.e., no significant changes in the breast occurred during follow-up).

### Pressure-controlled and force-controlled paddle

The pressure-controlled paddle (Sensitive Sigma Paddle, Sigmascreening) was installed on one mammography unit in February 2016. All 15 mammography technicians received the same application training by the vendor. After an introduction period, the paddle was implemented and exclusively used on one unit, while the second unit used the force-controlled paddle. The protocol for breast compression using a rigid force-controlled paddle was as follows: while carefully positioning the breast, the compression paddle is lowered until a force of 17–18 daN was reached or until the patient indicated intolerable pain. With the pressure-controlled paddle, the breast was compressed until the target pressure of 10 kPa was reached. Eight indicator LEDs on the paddle itself indicate the pressure qualitatively. The target pressure is reached when six LED’s light up.

The technicians were randomly working on both units. Starting from May 2016, exams performed with the pressure-controlled paddle were included.

### Data collection

The compression force, CBT, tube potential, tube current, anode and filter material, patient age, exam date, and laterality, were retrieved from the DICOM headers. The AGD was calculated for each exam by an in-house written software script (Python, Python Software Foundation) using the Dance model [10, 11] according to EUREF guidelines [1, 12].

The compression pressure could not be obtained directly, but it was determined by dividing the compression force by the breast-paddle contact area of the breast. The latter was estimated from the images using an in-house written script (Matlab, MathWorks) following the method described by de Groot et al [13].

### Data analysis

The follow-up examination pairs were divided in two groups: (1) both examinations being performed with the force-controlled paddle (“force-force group”) and (2) first examination performed with the force-controlled paddle and the second with the pressure-controlled paddle (“force-pressure group”). For each follow-up pair, the differences (exam 2–exam 1) in the CBT (ΔCBT [mm]), compression force (Δforce [daN]), compression pressure (Δpressure [kPa]), and AGD (ΔAGD [mGy]) were calculated. The differences in the parameters for the force-force group were taken as a reference for the intra-group variation when recompressing the same breast. Clinically relevant differences were defined as ΔCBT > ± 2 mm; Δforce > ± 2 daN; Δpressure > ± 1 kPa; and ΔAGD > ± 0.1 mGy (see supplemental material for more details).

### Pain score assessment

In an additional group of patients, pain experienced during the mammography exam was scored prospectively (period November 24, 2016–December 29, 2016). All consecutive patients, regardless of their indication, were randomly assigned to a mammography unit with either the pressure-controlled paddle or the force-controlled paddle. Patients were unaware that different paddles were used in our department, nor that a pain score would be assessed. After the complete examination, the technician asked the patient to score the intensity of pain experienced on a ten-point Numeric Rating Scale (NRS; 0 = no pain at all, 10 = worst pain imaginable). NRS pain scores ≥ 2 are considered to be clinically relevant [14, 15]. For this difference, to reach a power of (1-β) > 0.99 for an *α* = 0.05 and estimated standard deviation of ~ 2.5, a sample size of ≥ 2 × 58 patients would be required.

### Medical ethics approval

The local medical ethics committee approved both the retrospective and prospective part of the study. The necessity to obtain written informed consent was waived.

### Statistical analysis

Data was tested for normality (Shapiro-Wilk test), and reported as median and interquartile range (IQR), which is represented by [25th percentile, 75th percentile]. Differences between groups were tested using the Mann-Whitney *U* test. A subanalysis was performed by categorizing the exams according to mean CBT of each pair: ≤ 40 mm, 41–50 mm, 51–60 mm, 61–70 mm, ≥ 71 mm. Correlations were examined using a linear regression model and the correlation coefficient *R*^2^ was reported. All statistical analyses were performed using SPSS (IBM SPSS Statistics; version 23, IBM Corporation). Any *p* < 0.05 was considered statistically significant.

## Results

In total, 3188 patients were included having 3848 pairs of follow-up exams as some patients had two or three follow-up exams in the inclusion period. Table 1 shows the characteristics of the force-force and force-pressure groups. No clinically relevant differences were found between the two groups although CBT, time between follow-up, and AGD were significantly different.

**Table 1:** Details on the patient population in the force-force and force-pressure group. Values are reported as median [IQR] unless specified otherwise. The *p* values of the Mann-Whithney *U* test are reported. The median [IQR] values reported for age, CBT, contact area, compression force, compression pressure, and AGD are calculated from the mean value of exams 1 and 2 for each exam pair

	Force-force group		Force-pressure group		*p* value
Number of patients	2574		614		n.a.
Number of exam pairs	3234		614		n.a.
Age (years)	59	[52–67]	59	[53–68]	0.960
Time between follow-up (months)	12.2	[11.8–12.6]	12.2	[12.0–15.9]	* < 0.001
Mean number of follow-up exams [range]	1.2	[1–3]	1.0	[1]	n.a.
CBT (mm)	59	[50–67]	57	[49–65]	*0.001
Contact area (dm^2^)	0.62	[0.43–0.87]	0.63	[0.45–0.91]	0.123
Compression force (daN)	17	[16–18]	17	[16–18]	0.513
Compression pressure (kPa)	26	[19–38]	26	[18–37]	0.324
AGD (mGy)	1.64	[1.38–2.05]	1.57	[1.33–1.88]	* < 0.001

* Indicates significance

*n.a.* not applicable

**Fig. 1:**
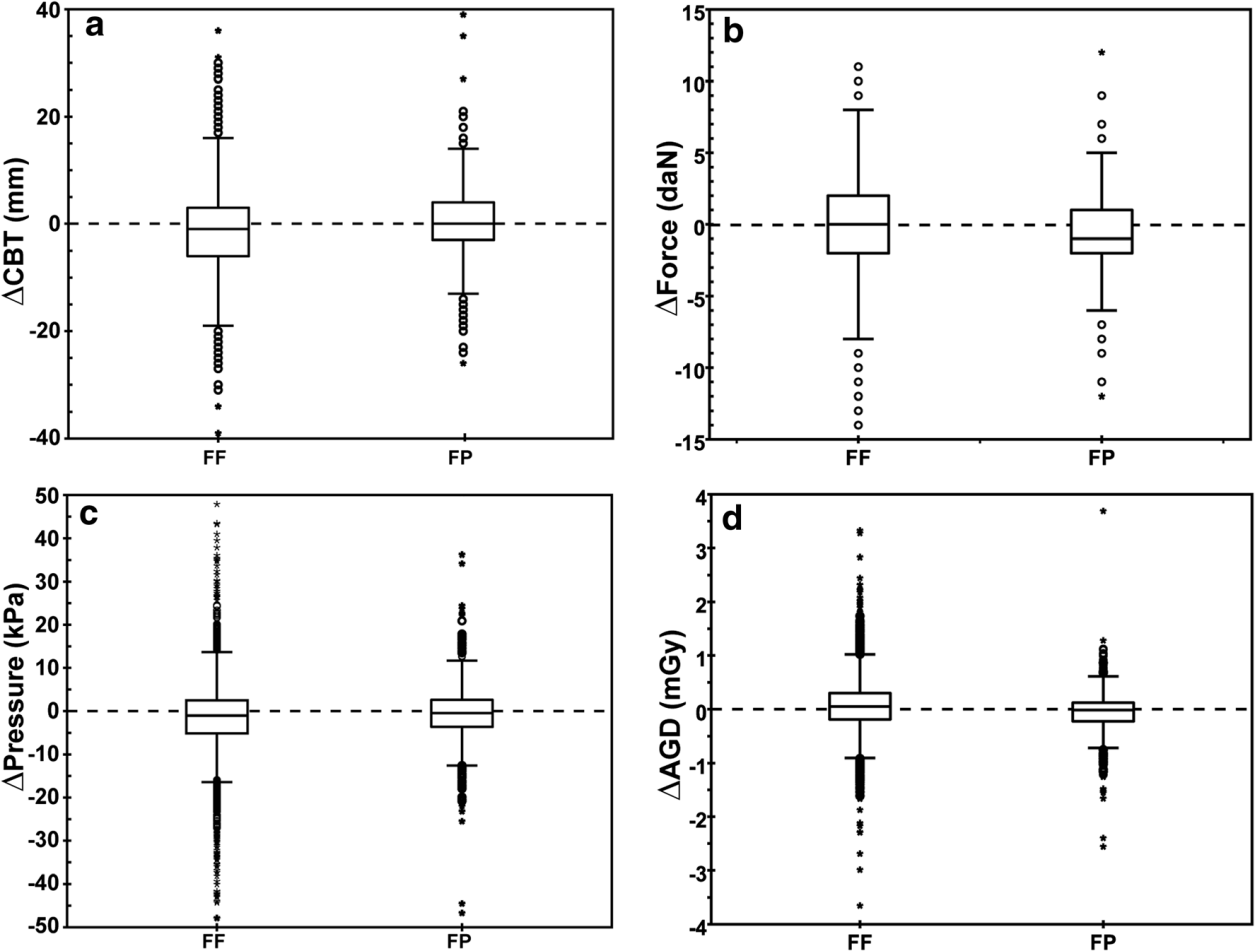
Comparison of differences (Δ) in (**a**) CBT, (**b**) force, (**c**) pressure, and (**d**) AGD between subsequent exam for the force-force (FF) and force-pressure (FP) group. For presentation purposes, the y-scale in **a**, **c**, and **d** was adjusted resulting in (**a**) 2 points, (**c**) 39 points, and (**d**) 5 points that were not shown

The median ΔCBT, Δforce, Δpressure, and ΔAGD between follow-up exams were close to zero for both groups (Fig. 1). Although statistically significant, no clinical relevant differences in the median values between the two groups were observed (Table 2).

**Table 2:** Median [IQR] values of the differences between exam pairs for the force-force and force pressure group for the whole study cohort and for the CBT subgroups. The *p* values of the Mann-Whithney *U* test are reported

	Force-force group		Force-pressure group		*p* value
All patients					
Number of exam pairs	3234		614		
ΔCBT (mm)	− 1.0	[− 6.0; 3.0]	0.0	[− 3.0; 4.0]	* < 0.001
ΔForce (daN)	0.0	[− 2.0; 2.0]	− 1.0	[− 2.0; 1.0]	*0.002
ΔPressure (kPa)	− 1.0	[− 5.1; 2.5]	− 0.5	[− 3.6; 2.6]	*0.005
ΔAGD (mGy)	0.05	[− 0.18; 0.30]	− 0.02	[− 0.22; 0.12]	* < 0.001
CBT ≤ 40 mm					
Number of exam pairs	252		66		
ΔCBT (mm)	− 1.0	[− 5.0; 3.0]	0.0	[− 3.0; 3.0]	*0.030
ΔForce (daN)	0.0	[− 3.0; 2.0]	− 1.0	[− 3.0; 1.0]	0.237
ΔPressure (kPa)	− 1.2	[− 6.6; 2.5]	0.0	[− 3.5; 3.1]	0.078
ΔAGD (mGy)	0.10	[− 0.12; 0.34]	0.01	[− 0.08; 0.11]	*0.010
CBT 41–50 mm					
Number of exam pairs	607		117		
ΔCBT (mm)	0.0	[− 3.0; 1.0]	0.0	[− 3.0; 3.0]	*0.048
ΔForce (daN)	− 1.0	[− 2.0; 1.0]	− 1.0	[− 3.0; 1.0]	0.430
ΔPressure (kPa)	− 1.5	[− 5.7; 2.2]	0.0	[− 4.4; 2.7]	*0.034
ΔAGD (mGy)	0.07	[− 0.11; 0.28]	− 0.04	[− 0.17; 0.09]	* < 0.001
CBT 51–60 mm					
Number of exam pairs	939		192		
ΔCBT (mm)	− 1.0	[− 6.0; 3.0]	1.0	[− 3.0; 4.0]	*0.001
ΔForce (daN)	0.0	[− 2.0; 2.0]	0.0	[− 2.0; 2.0]	0.839
ΔPressure (kPa)	− 1.1	[− 4.6; 2.3]	− 0.2	[− 3.3; 3.1]	*0.010
ΔAGD (mGy)	0.05	[− 0.14; 0.25]	− 0.05	[− 0.25; 0.09]	* < 0.001
CBT 61–70 mm					
Number of exam pairs	869		161		
ΔCBT (mm)	− 1.0	[− 6.0; 3.0]	0.0	[− 2.0; 5.0]	* < 0.001
ΔForce (daN)	0.0	[− 2.0; 2.0]	− 1.0	[− 2.0; 1.0]	0.159
ΔPressure (kPa)	− 0.7	[− 4.6; 2.4]	− 0.9	[− 3.3; 2.0]	0.415
ΔAGD (mGy)	0.02	[− 0.24; 0.30]	0.01	[− 0.27; 0.15]	0.126
CBT > =71 mm					
Number of exam pairs	567		78		
ΔCBT (mm)	− 1.0	[− 7.0; 4.0]	0.0	[− 4.0; 4.3]	0.186
ΔForce (daN)	0.0	[− 2.0; 2.0]	− 1.0	[− 3.0; 0.0]	* < 0.001
ΔPressure (kPa)	− 0.8	[− 5.7; 3.2]	− 1.7	[− 4.0; 0.7]	0.172
ΔAGD (mGy)	0.02	[− 0.36; 0.45]	0.01	[− 0.35; 0.25]	0.273

* Indicates significance

**Fig. 2:**
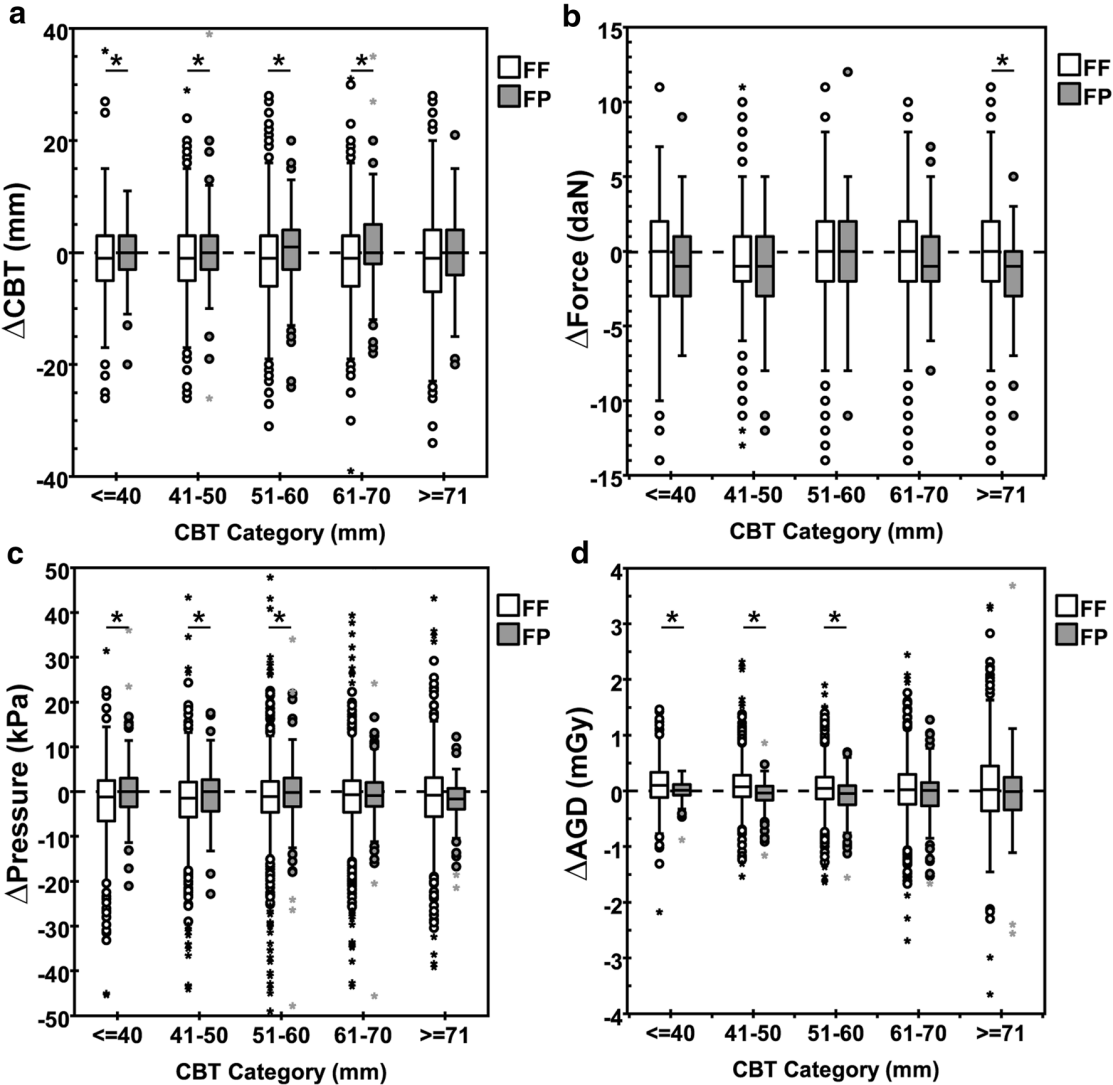
Comparison of differences (Δ) for different CBT categories in (**a**) CBT, (**b**) force, (**c**) pressure, and (**d**) AGD between subsequent exam for the force-force (FF) and force-pressure (FP) group. For presentation purposes, the y-scale in **a**, **c**, and **d** was adjusted resulting in (**a**) 2 points, (**c**) 39 points, and (**d**) 5 points that were not shown. * indicates significance

The subanalysis for CBT categories showed that the median ΔCBT, Δforce, Δpressure, and ΔAGD were close to zero across all CBT categories (Fig. 2). Although statistically significant in 10/20 instances, differences between the median values of both groups were clinically not relevant (Table 2).

**Fig. 3:**
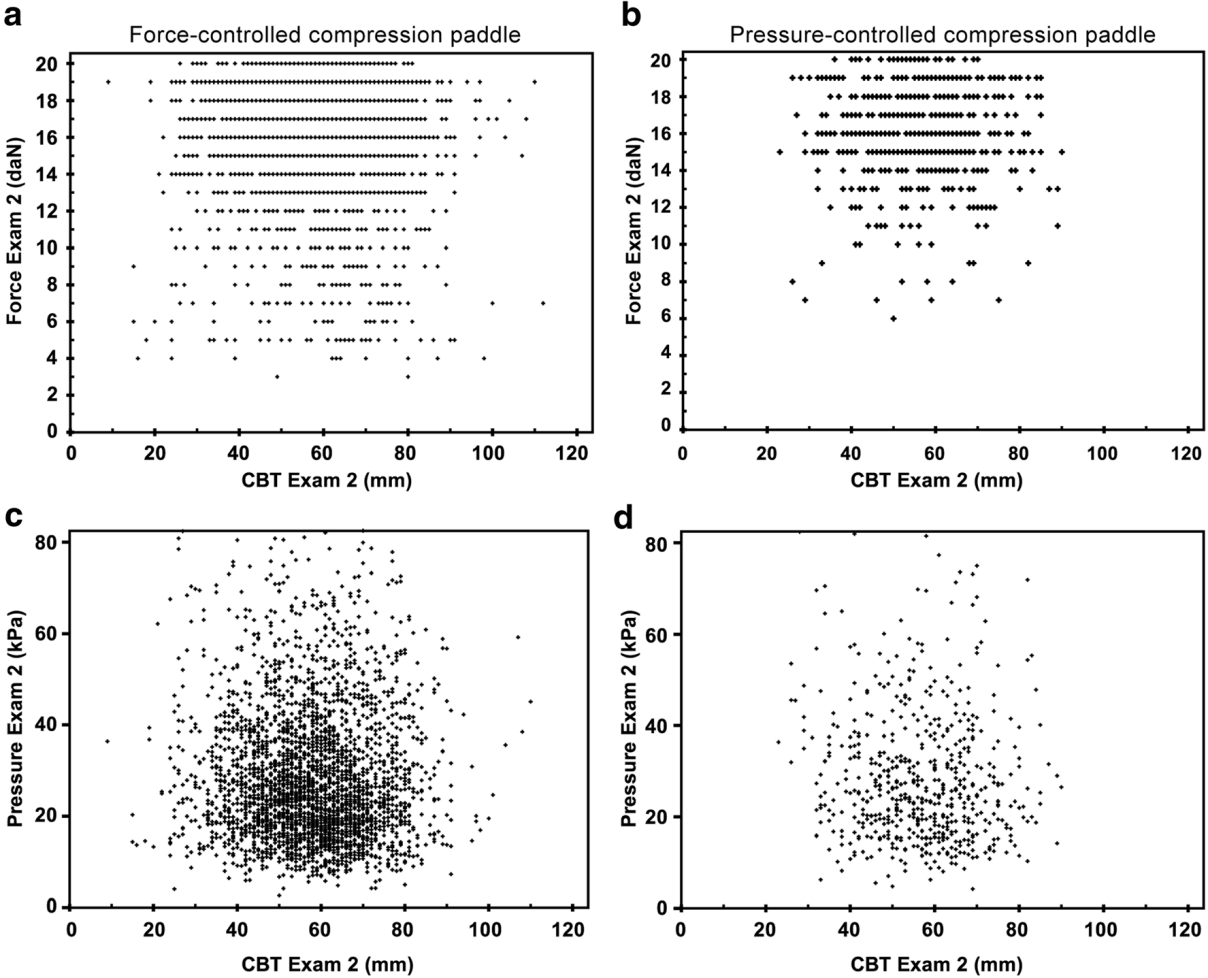
Compression force (**a**), (**b**) and pressure (**c**), (**d**) versus CBT of individual exams for the force-controlled paddle (left-hand side (**a**, **c**): second exam of each pair in the FF-group) and pressure-controlled paddle (right-hand side (**b**, **d**): second exam of each pair in the FP-group). The median [IQR] for the force-controlled paddle were force 17 [15; 18] daN; pressure 25.3 [18.2; 36.7] kPa; CBT 58 [49; 67] mm, and for the pressure-controlled paddle, force 16 [15; 18] daN; pressure 25.5 [19.1; 36.5] kPa; CBT 57 [49; 66] mm. No correlations were found (**a**, *R*^2^ < 0.001; **b**, *R*^2^ = 0.001; **c**, *R*^2^ = 0.008; **d**, *R*^2^ = 0.002)

Further analysis of the used compression force and pressure showed a large variation for both paddle types (Fig. 3), which may explain the observation that no clinical relevant median differences were found. As protocol dictated breast compression until a target force or pressure was obtained for the force- or pressure-controlled paddle respectively, it would have been expected that the applied force or pressure, respectively, were more or less constant irrespective of the CBT. However, no correlation was found between compression force or pressure and CBT (*R*^2^ ≤ 0.008). The large variation observed in Fig. 3 may also explain that for individual patients differences between the follow-up exams may reach clinically relevant levels. However, this is observed both for better or worse and in both the force-force and force-pressure group (Figs. 1 and 2).

**Fig. 4:**
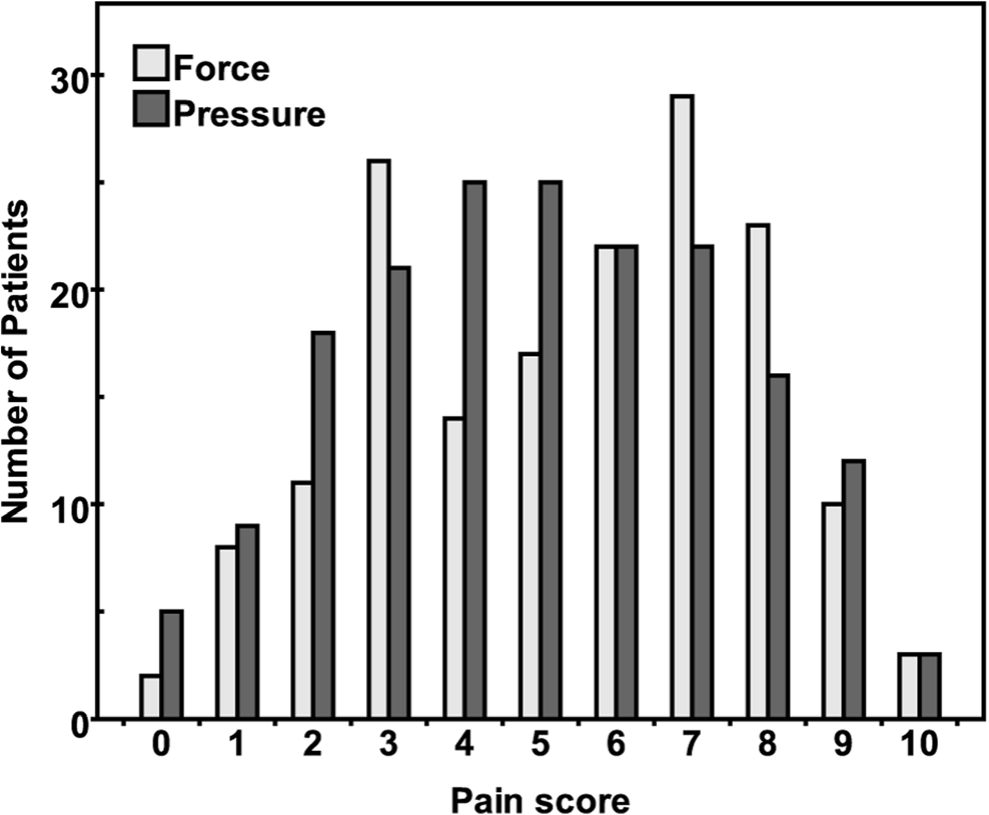
Distribution of pain scores using the numerical rating scale for examinations using either the force- or pressure-controlled paddle

The pain scores for exams performed with the force- (*n* = 165) or pressure-controlled paddle (*n* = 178) were not statistically different (*p* = 0.113, Fig. 4). The median [IQR] pain scores were 6 [3, 7] for the force-controlled paddle and 5 [3, 7] for the pressure-controlled paddle. The median [IQR] age and CBT was 57 [49–67] and 56 [49–65] years (*p* = 0.441) and 55 [46–65] and 57 [48–66] mm (*p* = 0.075) for the force- and pressure-controlled paddle group respectively.

## Discussion

In our study, we evaluated the pressure-controlled compression paddle with respect to the conventional force-controlled compression paddle in the everyday clinical practice of our institution. In contrast to prior studies in controlled experimental settings, we could not observe any clinically relevant added value of the pressure-controlled paddles. As such, we found no basis for preferring one paddle over the other.

Multiple studies were published by a single group showing the potential of the pressure-controlled paddle: a more standardized compression was feasible and discomfort and pain could be reduced, while the AGD was similar and image quality was not affected [4, 7]. However, the studies were performed in a highly-standardized setting, which raises the question of how the pressure-controlled paddle performs in clinical practice, where more variation in breast compression is present. A retrospective study evaluating the pressure-controlled paddle in a clinical setting for *n* = 39 patients found that the compression pressure of the pressure-controlled paddle is only weakly dependent on the breast contact area as opposed to the compression pressure of the force-controlled paddle [16]. However, whether clinically relevant differences in compression parameters between follow-up exams in favor of the pressure-controlled paddle could be observed was not studied. Our observations show a large spread in compression force, CBT, and AGD between follow-up exams, which was also found by Mercer et al [8, 9]. Moreover, significant differences existed in these parameters between technicians performing the compression.

The EUREF guidelines [1] merely indicate that “The breast should be properly compressed, but no more than is necessary to achieve a good image quality.” A higher compression force would lead to a smaller breast thickness which is believed to be beneficial for image quality, reduction of motion artifacts, and AGD. Although, a study of Holland et al [17] showed in a retrospective analysis of screening mammograms that applying too much or too low pressure lead to a reduced sensitivity and specificity, respectively. In clinical practice, achieving optimal breast compression is a tailor-made process in which technicians consider several factors, such as the patient’s pain tolerance and the risk of motion artifacts, as well as their experience.

There is a complex interplay between the actual discomfort/pain experience of the patient and obtaining a good breast compression. Influencing factors are patient characteristics, such as breast size and density, pain tolerance, and prior existing pain [2, 18], but also proper adjustment of the bucky/detector height [19], number of prior acquisitions within the exam [6], providing information about the examination [2, 20], and psychological factors which can be influenced by the social interaction between patient and technician [20, 21]. This might suggest that repeated training of the technicians, not only in terms of proper positioning but also patient communication, may be an important influential factor in reducing discomfort and pain while maintaining high image quality. As we could not observe any beneficial effects for the pressure-controlled paddles in the clinical setting, we recommend to further investigate the causes for the discomfort/pain experience because this is the basis for seeking solutions. Also analyzing more deeply the observed spread in compression parameters would enable to optimally deploy the potential of pressure-controlled breast compression in clinical practice.

Our study has some limitations. First, the applied pressure was not directly provided by either paddle. Therefore, the pressure was calculated by estimating the contact area from the images, which may deviate from the actual contact area. Despite this limitation, the Δpressure analysis confirms the findings of the ΔCBT and Δforce analysis. Second, there could have been changes in a breast between subsequent exams. To circumvent this, the BI-RADS score was 1 or 2 and equal for both exams. Furthermore, sufficiently large groups were studied to minimize the influence of cases where changes in the breast did occur. Third, we choose not to include an evaluation of the image quality for both paddles. Not finding any differences in compression parameters, we did not expect to observe a difference in image quality, and if so, we would not be able to attribute this to the paddle-type used. Fourth, we performed a single-center study, which might have led to bias in the selection of patients and technicians. It would be valuable to investigate the effect of the pressure-controlled paddle in the clinical practice of other institutions.

In conclusion, the evaluation of the pressure-controlled paddle in clinical practice revealed no clinically relevant differences in CBT, compression force, compression pressure, and AGD in comparison to the force-controlled paddle. The pain score assessment revealed no significant differences between the two paddles. As such, we found no basis for preferring the pressure-controlled paddle over the force-controlled paddle in our clinical practice.

## Electronic supplementary material

ESM 1 (DOCX 17 kb)

## Abbreviations

AGD: Average glandular dose
BI-RADS: Breast imaging-reporting and data system
BRCA: Breast cancer
CBT: Compresed breast thickness
CC-view: Craniocaudal view
DICOM: Digital imaging and communications in medicine
EUREF: The European Network of Reference Centres for Breast Cancer Screening
FF-group: Force-force group
FP-group: Force-pressure group
FFDM: Full-field digital mammography
IQR: Interquartile range
NRS: Numeric Rating Scale
PACS: Picture archiving and communication system
SPSS: Statistical Package for the Social Sciences
