# Supplementary material for: Evaluation of pressure-controlled mammography compression paddles with respect to force-controlled compression paddles in clinical practice
Source: Eur Radiol. 2019 Jan 7;29(5):2545–52. doi: 10.1007/s00330-018-5953-6 (PMC6443616; doi:10.1007/s00330-018-5953-6)
Supplement: Supplementary file 1 — (DOCX 17 kb) [file 330_2018_5953_MOESM1_ESM.docx]

**Supplemental Material**

*Clinically relevant differences in CBT, compression force, compression pressure and AGD*

As there were no studies in literature describing what ΔCBT and Δforce are noticable by patients, a reasonable assumption was made. Differences were considered clinically relevant when ΔCBT and Δforce were larger than ±2 mm and ±2 daN respectively, which corresponds to internationally established CBT and Force indicator accuracy criteria [1]. For the Δpressure the clinically relevant difference is calculated from the force indicator accuracy being ±2 daN / 20 daN (=maximal force) = ±10%, which results in ±1 kPa for a target pressure of 10 kPa. ΔAGD is considered clinically relevant for differences larger than ±0.1 mGy, considering the uncertainties in the AGD calculation and the very low cancer incidence risks related to 0.1 mGy[2]. Differences smaller than the values indicated here were considered to be clinically non-relevant.

1. European Communities (2013) European Guidelines for Quality Assurance in Breast Cancer Screening and Diagnosis. 4th ed. Supp. Luxembourg, Luxembourg: Office for Official Publications of the European Communities. Available via <http://www.euref.org/european-guidelines>. Accessed 7^th^ Nov 2018
2. Jeukens CRLPN, Lalji UC, Meijer E et al (2014) Radiation exposure of contrast-enhanced spectral mammography compared with full-field digital mammography. Invest Radiol 49:659-665
